# A synchronized daily electricity demand dataset for commercial and residential service areas of Dhaka, Bangladesh, with integrated meteorological and calendar attributes

**DOI:** 10.1016/j.dib.2026.113328

**Published:** 2026-10-14

**Authors:** MD Sajid Hasan, Md. Ashiqur Rahman, Sanjida Khanom, Mohammod Ashikur Rahman

**Affiliations:** Department of Computer Science and Engineering, Bangladesh University of Business and Technology, Rupnagar, Mirpur-2, Dhaka, 1216, Bangladesh

**Keywords:** Electricity demand forecasting, Residential and commercial energy consumption, Smart grid, Meteorological data, Open data, Dhaka, Bangladesh

## Abstract

There is limited publicly available data on electricity demand for South Asian cities, and utility records are usually not shared below the national level. This article provides a daily electricity demand dataset for two areas in Dhaka, Bangladesh: Mirpur, which is mainly residential, and Gulshan, which is mainly commercial. The demand records were obtained directly from Dhaka Electric Supply Company PLC (DESCO) with written permission. The data were obtained from the company’s daily control-room log. The log is maintained manually, with one sheet prepared for each calendar date. Each sheet contains the aggregate load of each distribution zone in megawatts (MW) at fixed times of the day. A continuous dataset was constructed by transcribing data from 597 separate sheets. Each individual reading was checked during the quality-control process. For each date, all logged readings were summed to obtain a single daily demand value. This produced a daily aggregate demand index in summed megawatts (MW).The dataset covers 597 consecutive days, from 1 January 2024 to 19 August 2025, for each study area. Six daily weather variables were obtained from the Open-Meteo Historical Weather API and matched with the corresponding observation dates. These variables include maximum, minimum, and mean temperature, rainfall, maximum wind speed, and sunshine duration. Each record also contains the day of the week, climatic season, public holiday, special holiday, and the name of any major national or religious event. Since the Bangladeshi holiday calendar is not available in a machine-readable format, these attributes were manually annotated using the official Bangladesh Gazette. The annotations were cross-checked before the dataset was released. Both areas were processed using the same procedure and covered the same period. Therefore, the two series are aligned by date and correspond to the same weather and calendar conditions. Within this common temporal framework, the two files represent different area-level electricity demand contexts. One area is mainly residential, while the other is mainly commercial. However, the dataset is not intended to provide a normalized comparison between residential and commercial electricity consumption. To facilitate further use of the dataset, it is provided as two CSV files. The dataset is openly available through Mendeley Data under a CC BY 4.0 licence. These data can support electricity demand forecasting, feature construction, unusual-pattern detection, and explainable machine learning. They are particularly useful for research in developing-country settings, where well-documented electricity demand data are often limited.

Specifications Table

**Table:** 

Subject	Energy
Specific subject area	Electricity demand forecasting, urban energy analytics, comparative residential and commercial electricity consumption datasets.
Type of data	Table (CSV), Figure, Chart; processed and annotated data derived from utility control-room records.
Data collection	Aggregate zone load readings in megawatts (MW) for the Mirpur and Gulshan distribution service areas of Dhaka, Bangladesh, were obtained from Dhaka Electric Supply Company PLC (DESCO) with written permission. The readings came from the company’s daily control-room records and were summed by date to obtain one daily demand value for each area (Table 7). Daily weather data, including maximum, minimum, and mean temperature, rainfall, maximum wind speed, and sunshine duration, were obtained from the Open-Meteo Historical Weather API. The weather data were collected for one reference point representing the study area and matched with the corresponding dates. The API endpoint was https://archive-api.open-meteo.com/v1/archive, with the Asia/Dhaka timezone. The full retrieval parameters are given in Table 8. Public holidays, government-declared special holidays, and major national or religious events were identified manually using the official Bangladesh Gazette holiday calendars. One daily demand value for each area was recorded as missing and was not imputed (Table 11, Table 12).
Data source location	Mirpur (residential distribution service area), Dhaka, Bangladesh; approximate centroid 23.8223° N, 90.3654° E. Gulshan (commercial distribution service area), Dhaka, Bangladesh; approximate centroid 23.7925° N, 90.4078° E.
Data accessibility	Repository name: Mendeley Data. Data identification number (DOI): 10.17632/3crvdfyvvp.1. Direct URL to data: https://data.mendeley.com/datasets/3crvdfyvvp/1. Instructions for accessing data: the complete datasets and the accompanying documentation are publicly available at the above DOI without restriction.
Related research article	None.

## 1 Value of the Data

• The dataset contains 597 consecutive daily electricity demand records from two distribution service areas in Dhaka: Mirpur, which is mainly residential, and Gulshan, which is mainly commercial. These data were not previously available publicly. Each record also includes the weather and calendar information for the same day. This allows users to study electricity demand along with the weather and calendar conditions of that day. Users do not need to collect and match these data separately.
• A key feature of this resource is that it allows the demand patterns of the two service areas to be studied side by side. The data provide a common basis for examining how electricity demand varies across the two areas. However, the dataset does not support a direct comparison of residential and commercial consumption intensity. Each service area may include different types of consumers. The dataset also does not provide information on consumer numbers, connected load, floor area, or service-area size. Therefore, a higher demand level in one area should not be interpreted as higher consumption intensity. Despite this limitation, the two series can be compared in terms of their daily demand patterns. Their common structure and observation period make such comparisons straightforward. This feature also distinguishes the resource from the publicly available electricity demand datasets summarized in Table 1. The data can be used for electricity load forecasting, energy demand prediction, feature engineering, unusual-pattern detection, smart-grid studies and machine-learning testing [1,2]. The matching weather and calendar information can also be used for explainable energy forecasting, where researchers can study how weather and time-related factors affect a prediction [3,4].

**Table 1:** Comparison of the present data with recently published open electricity demand datasets.

Dataset	Region	Period	Resolution	Weather	Holiday	Named events	Consumption context
Hossain et al. [5]	Bangladesh (national, divisional)	Nov 2019 – Dec 2024	Daily	Yes	Yes	No	National aggregate demand, generation and load shedding
Uceta-Acosta et al. [6]	Dominican Republic (national)	2021 – 2024	Hourly	Yes	Yes	No	National interconnected system demand
Monteiro et al. [7]	Portugal (Loureiro)	May 2022 – Sep 2023	15 min	Yes	–	–	172 residential buildings in one energy cooperative
Li et al. [8]	China (Guangxi)	2022 – 2023	Hourly	Yes	Yes	Extreme weather only	23 residential communities, transformer level
Liu et al. [9]	China (southern)	Multi-year	15 min	Yes	–	Extreme weather only	386 industrial and commercial consumers
This dataset	Bangladesh (Dhaka: Mirpur, Gulshan)	Jan 2024 – Aug 2025	Daily	Yes (6 variables)	Yes (2 types)	Yes (national and religious)	Two time-paired distribution service areas, predominantly residential and predominantly commercial, identical pipeline; not normalised by consumer count or connected load

“–” indicates that the attribute is not included in, or not reported for, that dataset.
• The dataset marks public holidays, special government holidays and major national and religious events, including Eid. This allows researchers to study how electricity demand changes during holidays and special periods, information that is not often included in open electricity demand datasets from South Asia.
• The data may also be useful for future studies on transfer learning and domain adaptation between residential and commercial demand. These uses are possible because the two datasets are paired, but they are not tested in this article.
• Electricity utilities, urban planners, policymakers and energy managers can also use the data for demand assessment, infrastructure planning, peak-demand management and energy policy planning for smart-city and smart-grid applications.
• The complete data, data dictionary and processing code are openly available under a CC BY 4.0 licence. This allows users to repeat the reported processing steps and reproduce the reported results.

## 2 Background

Good electricity demand data are important for research on power systems, energy management and demand forecasting. The need for such data has increased with growing cities and electricity use [1,2]. In recent years, more open demand datasets with weather and time-related information have become available [[5], [6], [7], [8]].

Openly available demand datasets are usually compiled at national, regional or city scale, or else at the level of a single consumption context such as a group of households or a set of industrial and commercial consumers [[5], [6], [7], [8], [9]]. As a consequence, the opportunity to contrast residential and commercial demand under identical environmental and calendar conditions remains limited, and it is particularly limited in developing-country settings such as Bangladesh, where the published work has concentrated on national aggregates or on single-city forecasting applications [5,10]. Weather, and especially temperature, is among the strongest exogenous drivers of electricity consumption and is therefore a necessary companion to any demand series [11,12].

Three obstacles explain why such datasets are uncommon in this setting, and each had to be addressed here. First, distribution-level demand is held by utilities as operational data and is not released as a matter of course; access required written permission covering not only academic use but also publication and public redistribution of the aggregated series. Second, the records are operational rather than archival: they are maintained manually as one control-room document per calendar date, recording zone load at fixed logging times, so a continuous series had to be assembled across the whole study period and screened for the transcription errors that manual records inevitably contain. Third, the contextual attributes that make demand data interpretable are not machine-readable in Bangladesh; public holidays, government-declared special holidays and major national and religious events were annotated by hand from the official Bangladesh Gazette and cross-checked before release. The resource described here is the outcome of resolving these three obstacles for two service areas over an identical 597-day window.

Recent work on electricity demand forecasting has moved from statistical baselines towards machine-learning and hybrid formulations, and the reported gains depend heavily on the exogenous attributes available to the model rather than on the learning algorithm alone [13,14]. Studies of intelligent energy systems and renewable-integrated networks report the same dependence, with weather and calendar attributes repeatedly identified as the decisive inputs at day-ahead and short-term horizons [[15], [16], [17]]. This literature makes the requirement on open data explicit: demand series are most useful when released together with synchronised environmental and contextual attributes and with a documented provenance for each field. The present resource is positioned against that requirement rather than against any particular modelling approach.

### 2.1 Positioning relative to existing open demand datasets

Table 1 positions the present data relative to recently published electricity demand datasets. The comparison covers region, observation period, temporal resolution, and whether weather, holiday and named-event attributes are included, so that the added value of the present data can be judged directly rather than inferred from the text.

### 2.2 Contribution of this data article

To address this gap, this article presents daily electricity demand data for two Dhaka distribution service areas with different main consumer types: Mirpur (mainly residential) and Gulshan (mainly commercial), covering 1 January 2024 to 19 August 2025. Demand data from DESCO were combined with weather data from the Open-Meteo archive [18,19] and with time-related information such as season, day of week, public holiday, special holiday and major national and religious events. Explainability is important in energy forecasting because it helps show which weather and time-related factors are linked to a prediction [3,4]. A dataset with these types of information can support such analysis.

The main contribution of this article is the dataset rather than a new method. The dataset combines four main features. To the authors’ knowledge, these features have not been brought together in an openly available electricity demand dataset for Bangladesh. These features are summarized in Table 1.

First, the dataset provides distribution-level demand records for Mirpur and Gulshan. Such records have not been readily available in public form. Previous studies in Bangladesh have mainly used national-level data. Some have focused on forecasting for individual cities. Second, the two demand series are matched by date. Both areas were processed using the same procedure. The data cover the same 597-day period. This allows the two series to be examined side by side. It also reduces differences caused by different study periods or processing methods. Third, each record includes major national and religious events. These include Eid festivals, Ramadan, Pohela Boishakh, and Victory Day. The dataset also includes two types of holiday flags. This information allows calendar and social events to be studied with weather-related changes in electricity demand. Fourth, the source and processing of the demand data are described in detail. This includes the original field and its unit. It also includes the aggregation method and completeness criterion. The treatment of records that were not directly observed is also reported.

The resource is intended as a reusable and auditable dataset. It can support electricity demand forecasting, feature construction, unusual-pattern detection, and explainable energy modelling. It is particularly relevant to developing-country settings.

The article does not propose or evaluate a new forecasting model. Instead, it describes the dataset and examines the usefulness of the released attributes for further analysis. The two service areas are not homogeneous by consumer type. The demand series are also not normalized by consumer count, connected load, or floor area. Therefore, differences between the two series should be understood as differences between the service areas. They should not be interpreted as direct differences between residential and commercial consumption.

## 3 Data Description

### 3.1 Dataset overview and construction workflow

The dataset comprises 597 daily electricity demand records per service area, collected from 1 January 2024 to 19 August 2025, for two electricity distribution service areas (EDSAs) of Dhaka: Mirpur (predominantly residential) and Gulshan (predominantly commercial). Both files were produced with the same collection, preprocessing, feature engineering and quality-checking procedure, so the two series are aligned in time and free of processing differences; the comparison they support is between two area-level demand contexts observed under the same environmental and calendar conditions. Each record contains the daily aggregate demand index together with weather attributes (maximum, minimum and mean temperature, rainfall, maximum wind speed, sunshine duration) and calendar attributes (day of week, climatic season, public holiday, special holiday, and the name of any major national or religious event). Meteorological attributes were obtained from the Open-Meteo Historical Weather API [18], which redistributes the ERA5 reanalysis [19], and are shared by both service areas; demand and contextual attributes were compiled from utility records and official calendars.

Fig. 1 shows the two study areas and the complete data construction workflow. The shaded areas show the mapped Mirpur and Gulshan localities within Dhaka. DESCO service-area boundaries are not publicly released. The figure also shows the 5.4 km distance between the service-area centroids, the meteorological reference point, and the ERA5 grid cell covering both areas. The basemap and area boundaries are based on OpenStreetMap. The figure also presents the five-stage workflow used to construct both released files. The same workflow was applied to both service areas, from demand acquisition and weather integration to feature generation, event annotation, quality control, and final data release. Thus, the figure provides a concise overview of the study areas and the steps used to prepare the released datasets.

**Fig. 1:**
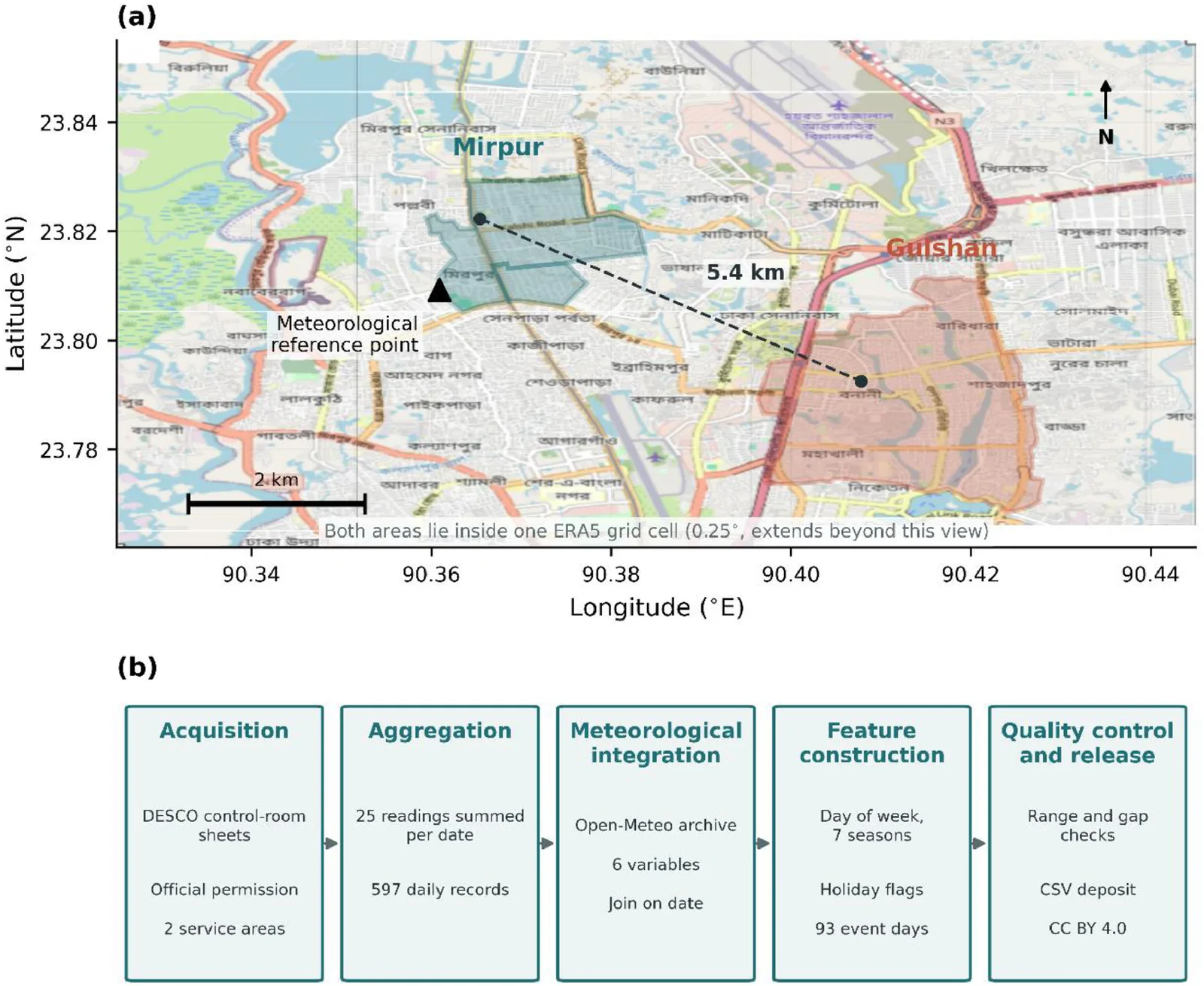
(a) Mirpur and Gulshan DESCO service areas in Dhaka. Shaded areas show the mapped localities; the dashed line indicates the 5.4 km centroid distance, the triangle shows the meteorological reference point, and the ERA5 grid cell covers both areas. © OpenStreetMap contributors. (b) Five-stage pipeline used to construct both files.

### 3.2 Variables and units of the released files

Table 2 defines every column of the two CSV files: the column name, data type, unit, meaning and a sample value. This table is intended to provide a clear and consistent reference for users working with the released datasets.

**Table 2:** Description of the variables included in the dataset.

Column name	Data type	Unit	Description	Sample value
Date	Date	MM/DD/YYYY	Date of the daily record.	1/1/2024

Total Demand	Numerical	MW (sum of readings)	Daily aggregate demand index for the service area, defined as the arithmetic sum of the 25 zone load readings (MW) logged by the DESCO control room on that date: hourly at 00:00 to 23:00, plus one additional reading at 19:30. Dividing by 25 returns the mean logged load for the day in MW. The index is proportional to, but not equal to, daily energy; As detailed in Section 4.1 and Table 7.	2734
Holiday	Categorical	–	Shows whether the date is a non-working day, comprising the weekly holidays (Friday and Saturday) and official public holidays declared in the Bangladesh Gazette. Dates carrying only a public-holiday declaration can be separated from weekly holidays using the Day_of_Week field.	Yes
Special Holiday	Categorical	–	Shows whether the date is a government-declared special holiday.	No
Max Temp	Numerical	°C	Highest air temperature of the day over the study area.	24.8
Min Temp	Numerical	°C	Lowest air temperature of the day over the study area.	15.4
Avg Temp	Numerical	°C	Average air temperature of the day over the study area.	20.1
Rain	Numerical	mm	Total rainfall during the day.	0
Max Wind Speed	Numerical	km/h	Highest wind speed of the day at 10 m above ground, as returned by the Open-Meteo archive in its default unit of kilometres per hour. The column header in the released CSV files reads “Max Wind Speed (m/s)”; this label is erroneous and the values are in km h⁻¹. The header will be corrected in a future version of the repository record.	10.3
Sunshine Duration	Numerical	hours	Total sunshine time during the day, changed from seconds to hours.	9.9
Day_of_Week	Categorical	–	Day of the week for the observation date.	Monday
Season	Categorical	–	Season assigned from the observation date.	Winter
Special_Event_Name	Categorical	–	Name of the major national or religious event on that date. “None” means no such event occurred. “None” is stored as a literal category label, not as a missing value. Software that converts the string “None” to a null token by default will display these records as empty; this conversion should be disabled when the file is read.	None

### 3.3 Temporal profile of daily electricity demand

Fig. 2 plots the two daily demand series over the whole study period, with 7-day moving averages and seasonal shading. It is included to show that coverage is continuous and that the two series follow the same seasonal cycle at different levels. Coverage is continuous across the study period, with the small number of missing demand observations resolved during preprocessing. Demand is higher in the warmer seasons and lower in winter in both areas. Mirpur generally records higher daily total demand than Gulshan throughout the period; this reflects the aggregate size and consumer composition of the two service areas and should not be read as a per-consumer or per-unit-floor-area comparison.

**Fig. 2:**
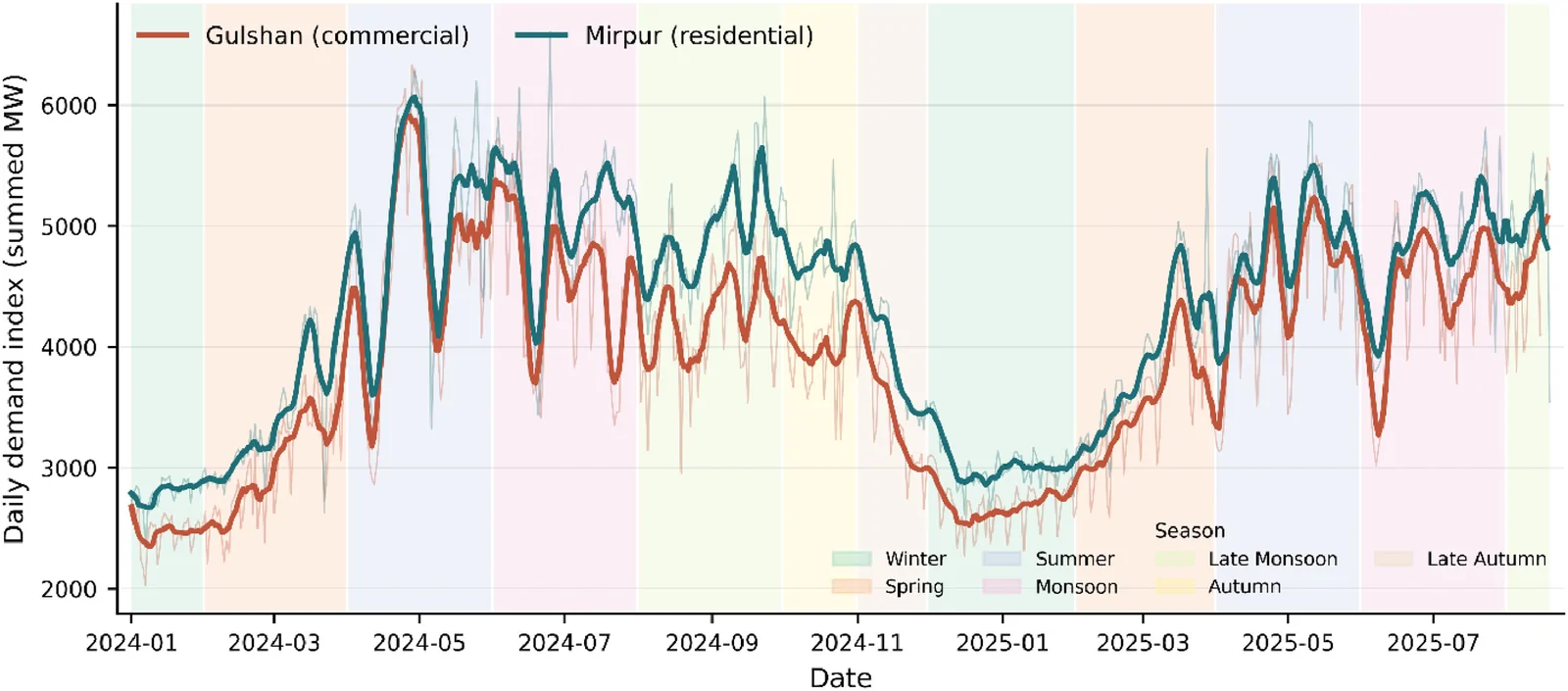
Daily electricity demand in Mirpur and Gulshan during the study period, together with 7-day moving averages and seasonal background shading.

### 3.4 Statistical characteristics of the recorded variables

#### 3.4.1 Summary statistics of demand and meteorological variables

Table 3 reports the mean, standard deviation, minimum and maximum of the demand and meteorological variables for both service areas. It gives the exact values that a re-user would otherwise have to estimate from the density plots, and it fixes the numerical range of every variable in the released files.

**Table 3:** Descriptive statistics of the demand and meteorological variables for the 597-day study period.

Variable	Unit	Mirpur (residential)				Gulshan (commercial)			
		Mean	SD	Min	Max	Mean	SD	Min	Max
Total demand	MW (sum of readings)	4315	947	2340	6608	3905	954	2022	6332
Maximum temperature	°C	31.1	3.9	20.4	40.8	31.1	3.9	20.4	40.8
Minimum temperature	°C	22.2	4.9	8.9	29.1	22.2	4.9	8.9	29.1
Mean temperature	°C	26.6	4.2	14.9	34.4	26.6	4.2	14.9	34.4
Rainfall	mm	5.1	11.9	0.0	174.7	5.1	11.9	0.0	174.7
Maximum wind speed	km/h	14.0	5.4	4.7	55.5	14.0	5.4	4.7	55.5
Sunshine duration	h	9.2	2.6	0.0	12.0	9.2	2.6	0.0	12.0

Demand is defined as the daily aggregate demand index described in Section 4.1. For each date, 25 zone load readings were recorded for each service area and summed to obtain a single daily demand value. The resulting value is expressed in summed megawatts (MW) and represents the total of the recorded zone loads for that day. Dividing this value by 25 gives the mean logged load for the day, which is 173 MW for Mirpur and 156 MW for Gulshan. The demand index is proportional to daily electricity demand but does not represent a direct measure of daily energy consumption in MWh. The statistical analysis is based on 596 demand observations for each service area. The demand record for 16 August 2025 is recorded as missing and is therefore excluded from the analysis. Similarly, 596 sunshine observations are used because the value recorded for 6 April 2024 was identified as invalid (Section 4.4). The same meteorological data are used for both service areas. These data were obtained from a single meteorological reference point shown in Fig. 1(a) and matched with the corresponding dates for both areas.

#### 3.4.2 Distributions of demand and meteorological variables

Fig. 3 shows the distribution of each variable summarized in Table 3. It is included because the shape of a distribution, in particular skewness in rainfall and bimodality in demand, is not visible from summary statistics alone. Temperature, rainfall, wind speed and sunshine duration all vary substantially across the study period, which gives the demand series a wide environmental envelope to be modelled against.

**Fig. 3:**
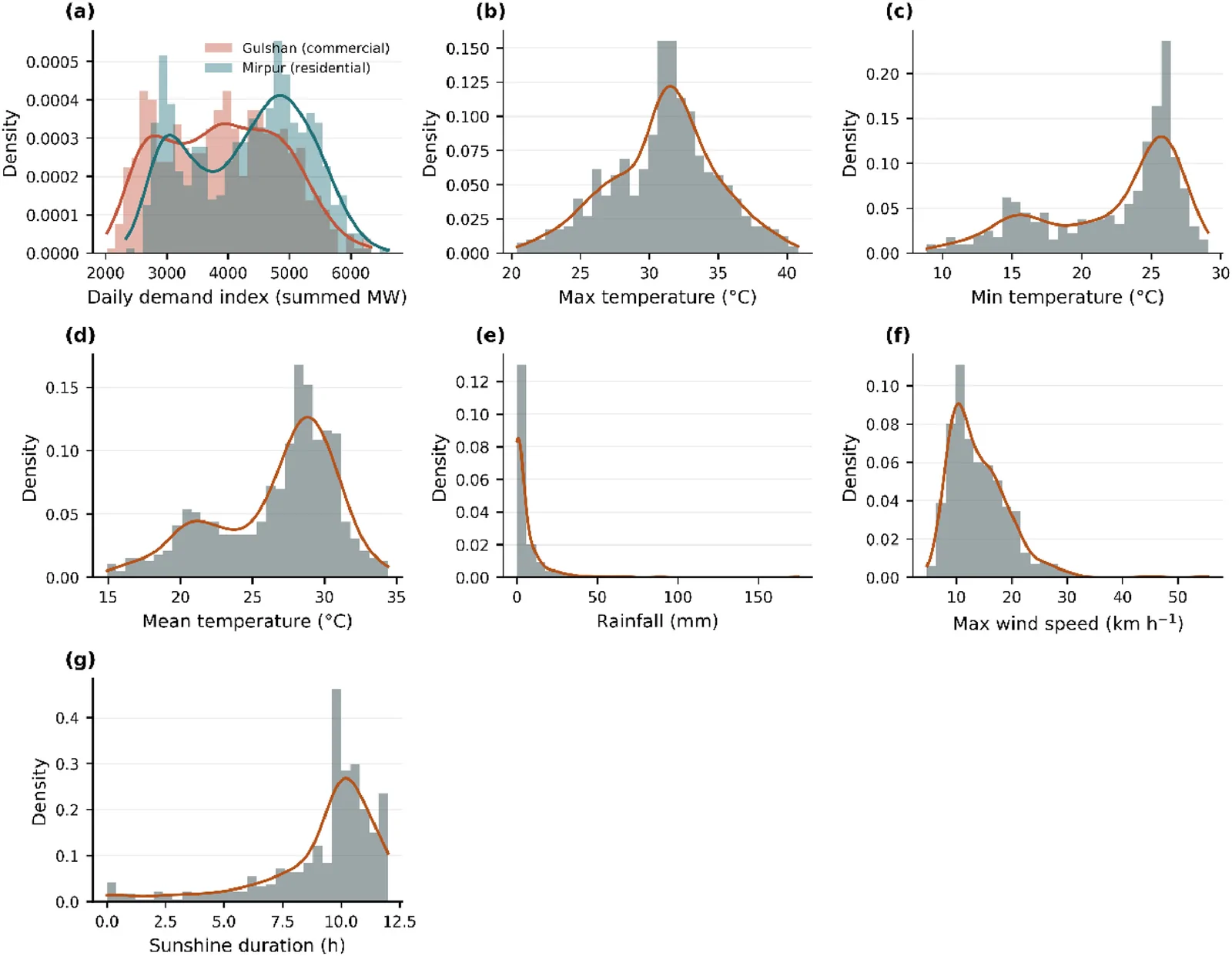
Distribution of daily electricity demand and meteorological variables: (a) electricity demand density for Mirpur and Gulshan; (b) maximum temperature; (c) minimum temperature; (d) average temperature; (e) rainfall; (f) maximum wind speed; and (g) sunshine duration.

#### 3.4.3 Seasonal and weekly demand characteristics

Table 4 groups the daily records by climatic season and by day of the week and reports the number of days and the mean and standard deviation of demand in each group. It quantifies the seasonal and weekly structure that the time series in Fig. 2 shows only visually. Demand is highest in the summer and monsoon seasons and lowest in winter in both service areas. Weekday means are comparatively stable, with the Friday weekend effect visible in both areas.

**Table 4:** Mean daily aggregate demand index (summed MW) by climatic season and by day of the week.

Group	Days	Mirpur (residential)		Gulshan (commercial)	
		Mean	SD	Mean	SD
By climatic season					
Winter	93	2948	199	2621	217
Spring	119	3659	596	3269	535
Summer	122	4921	712	4651	785
Monsoon	122	4985	551	4552	665
Autumn	31	4724	334	4047	398
Late autumn	30	3966	470	3475	497
Late monsoon	79	4915	481	4334	540
By day of the week					
Sunday	85	4334	950	4026	988
Monday	86	4328	964	4008	973
Tuesday	86	4376	943	4079	967
Wednesday	85	4381	944	4055	987
Thursday	85	4355	954	4031	967
Friday	85	4128	931	3441	754
Saturday	84	4304	951	3695	861

#### 3.4.4 Calendar coverage and day-to-day variability

Fig. 4 presents the temporal structure of daily electricity demand in the two service areas. It shows the same daily demand values in three ways. First, the seasonal distributions show how demand varies across the climatic seasons. Second, the weekly averages show differences in demand across the days of the week, including the Friday variation from the Sunday–Thursday baseline. Third, the calendar heat maps show the daily demand values for Gulshan and Mirpur, with one cell for each date. These heat maps provide a visual check of the 597-day coverage and show day-to-day variation in demand. Any missing record within the study period would appear as a blank cell. The blank cells after 19 August 2025 are outside the study period and do not represent missing observations.

**Fig. 4:**
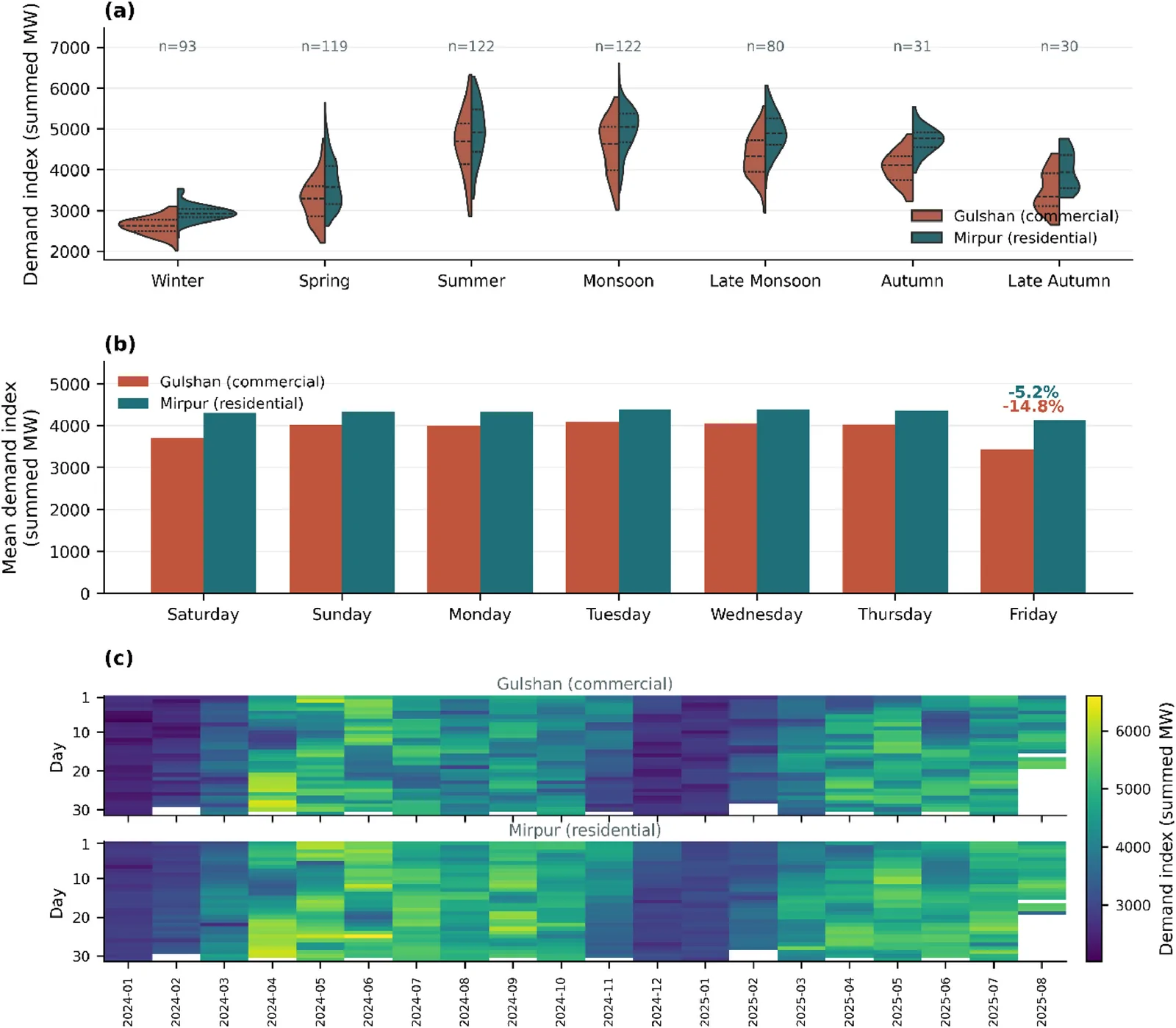
Temporal patterns of daily demand. (a) Seasonal distribution. (b) Weekly variation. (c) Daily demand heat maps for Gulshan and Mirpur.

### 3.5 Relationships between demand and the contextual attributes

#### 3.5.1 Association with the meteorological variables

Fig. 5 shows how electricity demand is related to the available explanatory variables. It is included to demonstrate that the weather attributes carry usable signal, which is the main condition for reusing the data in forecasting and explainable modelling. The Pearson correlation matrices for Mirpur and Gulshan show a stronger positive relationship between demand and temperature-related variables. Rainfall, sunshine duration, holidays, weekends and wind speed show weaker relationships. The right panel shows a positive relationship between mean daily temperature and demand in both areas. These results describe the data only; they do not prove that these variables cause changes in electricity demand.

**Fig. 5:**
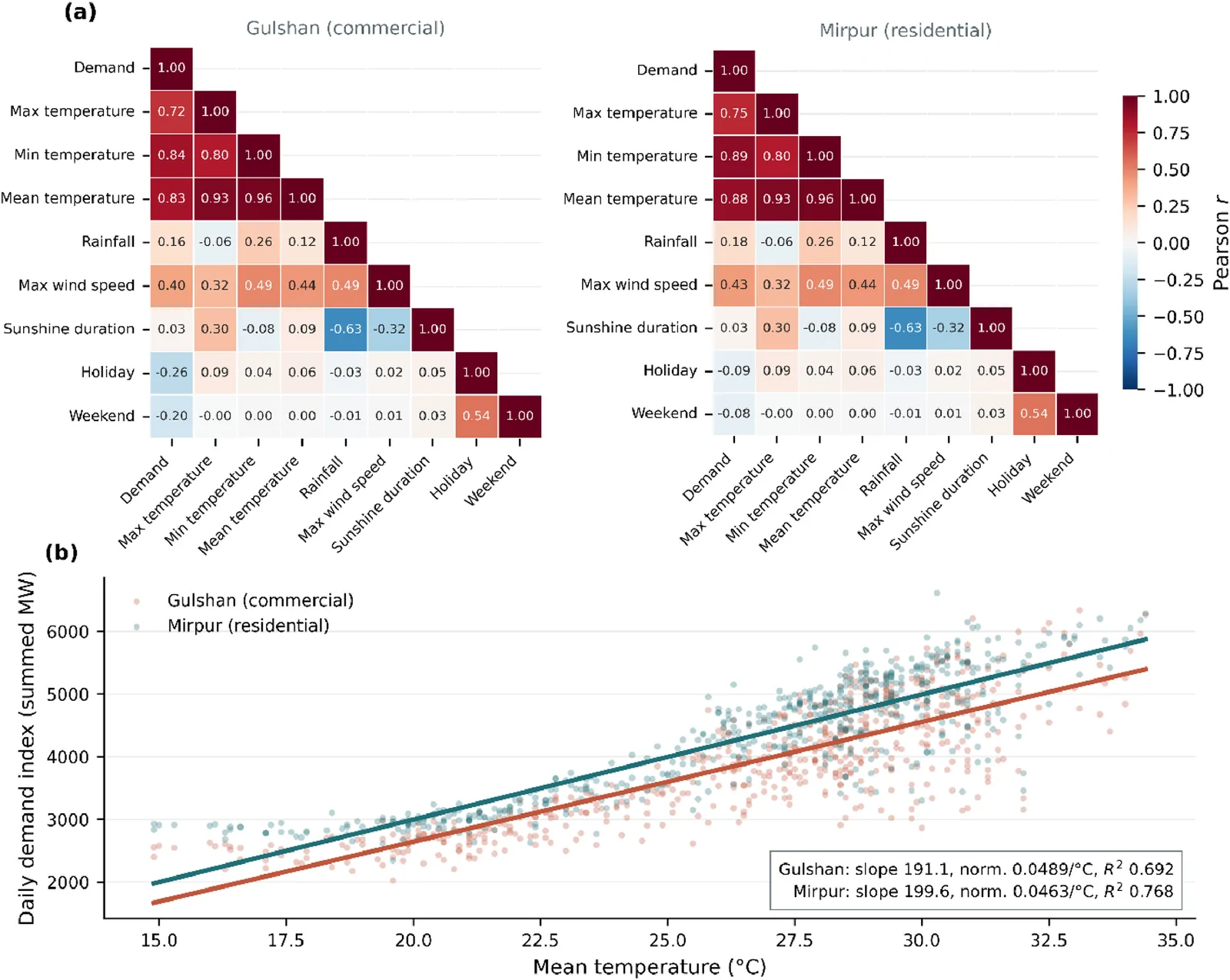
Relationships between electricity demand and explanatory variables: Pearson correlation matrices for Mirpur (left) and Gulshan (centre), and linear relationships between average temperature and electricity demand with fitted regression lines (right).

#### 3.5.2 Demand during public holidays and major events

Fig. 6 summarizes the behavior of the demand series around public holidays and major national or religious events, expressed as percentage deviation from the local baseline. It is included because the holiday and event annotations are a distinguishing attribute of this dataset, and the figure shows that those annotations correspond to measurable changes in demand. The deviations differ between the two areas, and longer holiday periods are generally associated with larger negative deviations.

**Fig. 6:**
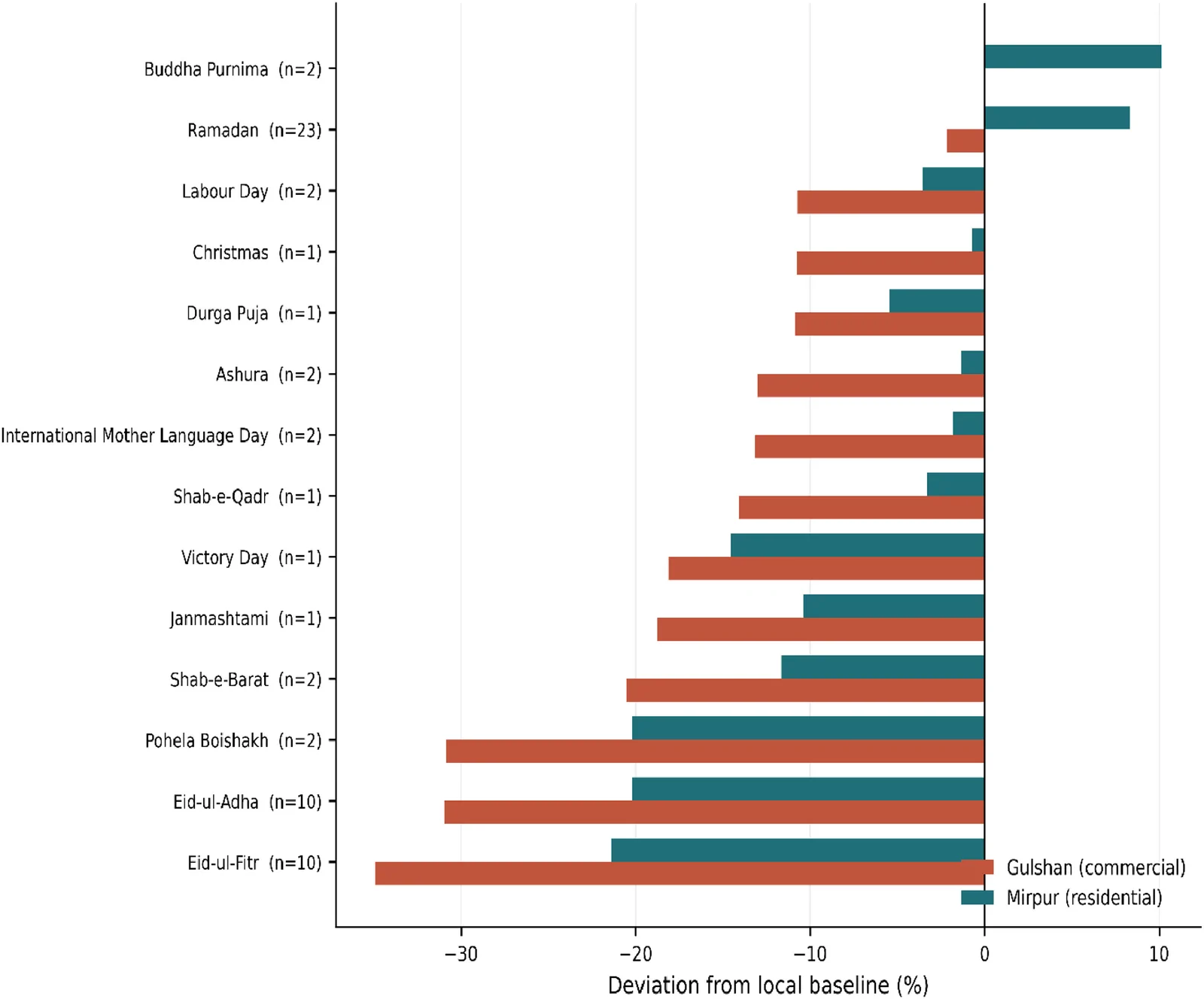
Percentage deviation in demand during public holidays and major national and religious events for Mirpur and Gulshan. Numbers indicate the dates for each event.

### 3.6 Comparison of the residential and commercial service areas

#### 3.6.1 Distributional and weekly comparison of the two series

Fig. 7 compares the two demand series. It is included because the paired residential–commercial structure is the central contribution of this dataset, and this figure shows what that pairing makes visible. The overall demand levels are different, but the daily percentage changes show similar variation in the two areas.

**Fig. 7:**
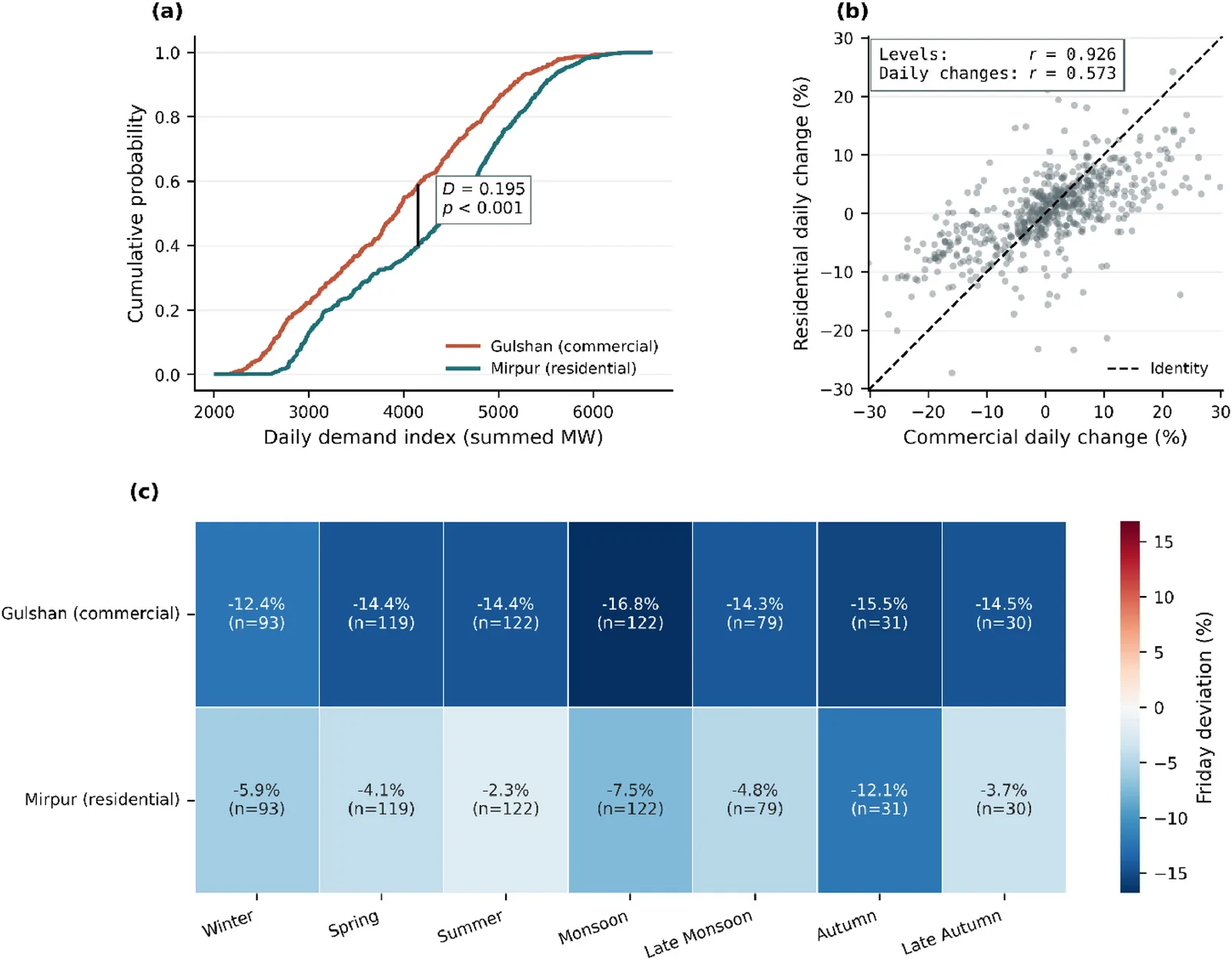
Statistical comparison of residential and commercial electricity demand: (a) empirical cumulative distribution functions of daily demand; (b) relationship between daily percentage changes in Mirpur and Gulshan; and (c) seasonal Friday demand deviation relative to the Sunday–Thursday baseline.

#### 3.6.2 Demand ratio and short-term variability

Table 5 reports the same comparison numerically. It gives the distribution of the Mirpur-to-Gulshan demand ratio and of the absolute day-to-day percentage change in each area, so that the stability of the ratio and the difference in short-term variability can be quoted as values rather than read from a plot. The residential-to-commercial demand ratio is relatively stable across the study period, and the distribution of absolute day-to-day percentage change indicates different short-term variability in the two areas.

**Table 5:** Comparative demand dynamics of the two service areas over the 597-day period.

Quantity	Mean	SD	Median	Min	Max
Mirpur-to-Gulshan demand ratio (−)	1.115	0.099	1.113	0.649	1.690
Absolute day-to-day change, Mirpur (%)	5.15	5.05	3.74	0.00	39.38
Absolute day-to-day change, Gulshan (%)	7.99	7.04	5.93	0.02	44.73

### 3.7 Data completeness and quality assessment

Table 6 reports the completeness of each variable after preprocessing and identifies the source of each field. It also indicates the two records that were retained as missing. The Special_Event_Name field contains the name of a major national or religious event when applicable. For other dates, it is recorded as “None”. Thus, this field has no missing values. The quality checks included several steps. Values were checked against physically plausible ranges. The 597-day study period was checked for continuity. Duplicate dates were checked, and dates were aligned across the two service areas. The completeness of the released fields was also assessed.

**Table 6:** Completeness of each variable in the released datasets (n = 597 records per service area).

Variable	Records	Complete (%)	Note
Date	597	100.0	Continuous daily calendar, no gaps or duplicates.
Total Demand	596	99.83	One value (16 August 2025) released as missing rather than imputed; As described in Table 11, Table 12.
Max Temp	597	100.0	Retrieved from Open-Meteo archive.
Min Temp	597	100.0	Retrieved from Open-Meteo archive.
Avg Temp	597	100.0	Retrieved from Open-Meteo archive.
Rain	597	100.0	Retrieved from Open-Meteo archive.
Max Wind Speed	597	100.0	Retrieved from Open-Meteo archive.
Sunshine Duration	596	99.83	Retrieved from Open-Meteo archive. One value (6 April 2024) is an out-of-range placeholder and should be treated as missing; As described in Section 4.4.
Holiday	597	100.0	Derived from the official Bangladesh Gazette calendar.
Special Holiday	597	100.0	Derived from government special-holiday notifications.
Day_of_Week	597	100.0	Derived deterministically from Date.
Season	597	100.0	Derived deterministically from Date.
Special_Event_Name	597	100.0	“None” on 504 dates without a major event.

Together, the released files provide a checked and well-documented dataset for comparing residential and commercial electricity demand. The demand, weather, calendar and event information can be reused for demand forecasting, explainable artificial intelligence, unusual-pattern detection, smart-grid studies and urban energy research. The paired structure may also support future work on transfer learning and domain adaptation. All of these applications operate at daily resolution. Because the released series are daily aggregates, the data cannot support intraday load-shape analysis, peak-hour forecasting or ramping studies.

## 4 Experimental Design, Materials and Methods

The dataset was developed through a five-stage pipeline. The stages include demand acquisition and aggregation, meteorological data integration, temporal and contextual feature construction, preprocessing and quality control, and final data assembly and release. The complete pipeline is presented in Fig. 1. The same procedures were applied to both service areas.

### 4.1 Study areas and electricity demand acquisition

Electricity demand data were collected for two electricity distribution service areas (EDSAs) in Dhaka, Bangladesh: Mirpur and Gulshan. DESCO records the electricity load of each distribution zone separately. Mirpur and Gulshan are two of the six distribution zones covered by these records. Mirpur mainly serves residential consumers, while Gulshan mainly serves commercial consumers. However, both areas also include some other consumer categories. Therefore, Mirpur and Gulshan are described based on their dominant consumer mix rather than as fully residential or commercial areas. DESCO does not provide consumer counts, connected load, floor area, or detailed sector composition for these zones. As a result, the demand data cannot be normalised using these factors. The data are therefore presented as area-level aggregates. The characterisation of each area rests on the established land use of the two zones rather than on a consumer census, which DESCO does not release. Gulshan is one of the principal commercial and diplomatic districts of Dhaka, containing a high concentration of offices, embassies, hotels and retail premises, whereas Mirpur is a densely populated residential district developed primarily as housing. The labels are therefore descriptive of the predominant land use of each service area and are used in that sense throughout this article. DESCO provided the original records under written permission for academic research, publication, and public sharing of the aggregated daily data. The records cover the period from 1 January 2024 to 19 August 2025. This provides 597 daily observations for each area.

The records supplied by DESCO come from the company’s daily control-room document, titled “Maximum Demand, Supply, Load shed, L/S Feeders information at a glance”. One sheet is prepared for each calendar date.

Each sheet contains one row for each distribution zone. The two zones used in this study are labelled “Gulshan (Total)” and “Mirpur (Total)”. Each sheet also contains columns for the recorded times under the row header “Control/Time”. The recorded quantity is the aggregate load of each zone, expressed as an integer in megawatts (MW).Readings are recorded hourly from 00:00 to 23:00. An additional reading is recorded at 19:30 to capture the evening peak. Thus, there are 25 readings for each zone on each date.

The released daily variable is calculated by summing the 25 readings recorded for that zone and date, D(d) = Σ P(d,k) for k = 1 to 25, where P(d,k) is the k-th logged zone load in megawatts. The 19:30 reading overlaps with the 19:00–20:00 interval. Therefore, the sum does not represent an exact daily energy total. The variable is released as a daily aggregate demand index in summed megawatts rather than in MWh. The same calculation is applied to every date and to both service areas. This provides a consistent daily measure for forecasting, seasonality, event effects, and comparative analyses. Because the index is based on a fixed number of readings, dividing it by 25 gives the mean logged load for that date in MW. Users who require a physical load measure can therefore use this mean daily load. An approximate daily energy value in MWh can be obtained by excluding the duplicated evening-peak reading. This reading accounts for roughly 5% of the reported value.

The released series were checked against the original source documents. Each daily control-room sheet contains the system total for each logging time, together with the individual zone readings. The zone readings were checked against the corresponding system totals. This provided an internal check for possible transcription errors.This reconciliation was performed using the source sheets as a verification step. Readings for the other distribution zones and the system totals are covered by the data-sharing permission but are not included in the released dataset.

A date was retained only when the complete set of readings was available for the relevant zone. Dates with any missing, blank, or invalid reading were treated as missing at the daily level and handled as described in Section 4.4. No individual consumer, meter, or premises data were accessed. The smallest spatial unit in the dataset is the service area.The resolution of the released data follows the terms under which the data were obtained. DESCO granted written permission for academic use, publication, and public redistribution of the aggregated daily series for the two study areas. The underlying control-room records remain the property of the utility and are not covered by this permission. Therefore, they cannot be deposited.

Daily aggregation is the finest resolution at which this resource can be released. It is not a modelling choice. To support reproducibility, the provenance of the demand variable, the aggregation equation, and the completeness criterion are documented in Table 7. A reuser with control-room input of the documented structure can therefore reproduce the released series exactly.

**Table 7:** Provenance of the released daily demand variable.

Item	Value
Source document	DESCO daily control-room record, “Maximum Demand, Supply, Load shed, L/S Feeders information at a glance”
Distribution	One spreadsheet per calendar date, supplied by the DESCO control room under written permission
Rows used	Gulshan (Total) and Mirpur (Total), two of the six zone rows recorded on the sheet
Recorded quantity	Aggregate load of the distribution zone
Unit of the original field	MW, recorded as an integer
Logging times	Hourly 00:00 to 23:00, plus one additional reading at 19:30 (25 readings per date)
Aggregation equation	D(d) = Σ P(d,k), k = 1 to 25
Unit of the released field	MW (sum of readings); daily aggregate demand index
Recovery of a physical quantity	D(d) / 25 gives the mean logged load for the date in MW
Completeness criterion	A date is retained only if all readings for that zone are present and valid; otherwise the day is released as missing
Records released as missing	16 August 2025, both service areas (Table 11, Table 12)
Internal verification	Zone rows checked against the system total recorded on the same source sheet; the source values themselves are not released

### 4.2 Meteorological data retrieval and integration

Daily meteorological data for the same period were obtained from the Open-Meteo Historical Weather API [18]. The API provides weather data based on the ECMWF ERA5 reanalysis [19]. Six daily weather variables were collected: maximum, minimum, and mean 2 m air temperature, precipitation, maximum 10 m wind speed, and sunshine duration. A single meteorological reference point was used for the study area, as shown in Fig. 1(a). The same weather series was then used for both service areas. The centroids of Mirpur and Gulshan are about 5.4 km apart. Both areas also fall within the same ERA5 grid cell. ERA5 data are provided on a regular grid of about 0.25 degrees, which is approximately 28 km at this latitude. Therefore, using either centroid would return the same weather values. The weather variables in the two released files are thus identical by construction. A separate request for the second centroid would not provide a different series. Sunshine duration was returned in seconds and converted to hours. Wind speed was returned in kilometres per hour (km h⁻¹), which is the API default. No unit parameter was specified in the request. Therefore, the values are reported in km h⁻¹. The released column header incorrectly states m/s. The weather data were then merged with each demand dataset using the observation date. An inner join was used and checked to retain all 597 dates.

Weather is included in the dataset as a shared covariate for both service areas. This allows users to examine the relationship between weather and demand within each area. It also allows the two demand series to be compared under the same weather conditions. However, the data cannot capture local weather differences between Mirpur and Gulshan. Local differences in temperature, rainfall, or wind may exist between the two areas. These differences are not represented in the released data. Therefore, any difference in demand response to weather should not be attributed to local microclimatic conditions. Separate weather stations in the two service areas would be needed to study such differences. Such observations were not available for the study period. Fig. 1(a) shows the two service areas and the ERA5 grid cell covering both areas. This also explains why a single weather series was used for both areas and why the dataset cannot capture microclimatic differences between them.

#### 4.2.1 Retrieval parameters

Table 8 lists the exact API request used. It is included so that the meteorological columns can be regenerated identically by any reuser; reissuing this request reproduces those columns exactly.

**Table 8:** Open-Meteo Historical Weather API retrieval parameters.

Parameter	Value
Service	Open-Meteo Historical Weather (Archive) API [18]
Endpoint	https://archive-api.open-meteo.com/v1/archive
Underlying reanalysis	ECMWF ERA5 / ERA5-Land [19]
Retrieval coordinates	latitude= 23.8093, longitude= 90.3609; one reference point for the study area, applied to both service areas
Start date	start_date=2024-01-01
End date	end_date=2025-08-19
Daily variables	temperature_2m_max, temperature_2m_min, temperature_2m_mean, precipitation_sum, wind_speed_10m_max, sunshine_duration
Units	temperature_unit=celsius, precipitation_unit=mm; wind speed returned in the service default unit of km/h, no wind_speed_unit parameter being set; sunshine duration converted from seconds to hours
Timezone	timezone=Asia/Dhaka
Access tier	Free non-commercial API
Date of retrieval	2025-10-22

### 4.3 Temporal and contextual feature construction

Day of week was derived deterministically from the observation date. Climatic season was assigned from the calendar month of the observation date using the fixed partition given in Table 9, which follows the conventional Bangladeshi seasonal cycle approximated to whole calendar months. The assignment is deterministic and applies no meteorological threshold, so it can be reproduced exactly from the date column alone. Public holidays and government-declared special holidays were annotated manually from the official holiday calendar published in the Bangladesh Gazette by the Government of Bangladesh for 2024 and 2025, together with subsequent special-holiday notifications. Major national and religious events, including the Eid festivals, were annotated by name in the Special_Event_Name field from the same official sources. Every annotated date was cross-checked independently against the published calendar before release. These attributes allow demand variation associated with social and institutional time to be studied alongside weather-driven variation.

**Table 9:** Definition of the seven climatic season categories and the number of records in each over the study period.

Season	Calendar months	Records
Winter	December – January	93
Spring	February – March	119
Summer	April – May	122
Monsoon	June – July	122
Late Monsoon	August – September	80
Autumn	October	31
Late Autumn	November	30

The record counts are unequal because the observation window begins on 1 January 2024 and ends on 19 August 2025, so the months of the second year are not all represented. The counts sum to the 597 records of each file.

### 4.4 Preprocessing, imputation and quality checking

Before final assembly, both datasets were checked for duplicate dates and missing values. The dates were also checked for malformed or out-of-range entries. Each meteorological variable was then screened against an explicit range of physically plausible values, listed in Table 10. A value falling outside its range was treated as invalid rather than corrected, so that no substituted value enters the released series. The bounds are deliberately wide: they are set to identify placeholder values and transcription faults rather than to exclude genuine extremes, and the sunshine bound corresponds to the maximum astronomical daylight at the latitude of Dhaka. One daily demand observation is missing for each service area. Both missing observations occur on 16 August 2025, representing 0.17% of the records. The date was a Saturday and was also flagged as a public holiday. It was associated with the named event Janmashtami. Therefore, simple neighbour-based imputation was not considered reliable for this date. The previous day was Friday, the weekly holiday, while the following day was an ordinary working Sunday. The demand patterns of these adjacent days may therefore differ from the missing date.

**Table 10:** Physically plausible ranges applied during quality control.

Variable	Accepted range	Basis
Maximum temperature	0 – 50 °C	Beyond the recorded extremes for Dhaka
Minimum temperature	0 – 40 °C	Beyond the recorded extremes for Dhaka
Mean temperature	0 – 45 °C	Beyond the recorded extremes for Dhaka
Rainfall	0 – 500 mm	Beyond the highest recorded daily rainfall
Maximum wind speed	0 – 120 km h⁻¹	Beyond the highest recorded gust
Sunshine duration	0 – 13.5 h	Maximum astronomical daylight at this latitude

For this reason, the record is released as an empty cell rather than being imputed. Imputation would introduce an estimated value into a dataset intended for modelling. The estimate is also sensitive to the imputation rule. Table 12 shows that four reasonable rules produce values that differ by up to 740 index units for this date. The value obtained from the neighbour-mean rule is provided in Table 11 for users who prefer to fill the missing value. Users who prefer complete-case analysis can also remove the date. Either approach changes the series mean by only about 0.03%. Therefore, the results reported in this article are not affected by the treatment of this missing value.

**Table 11:** Missing daily demand observations and the treatment applied.

Service area	Observations	Missing	Missing (%)	Treatment
Mirpur (residential)	597	1	0.17	Released as missing rather than imputed. The value given by the neighbour-mean rule, for users who prefer to fill it, is 5142; As Described in Table 12.
Gulshan (commercial)	597	1	0.17	Released as missing rather than imputed. The value given by the neighbour-mean rule, for users who prefer to fill it, is 4628; As Described in Table 12.

The affected date is 16 August 2025 in both service areas. The record is released as an empty cell so that users may apply their own treatment; Table 12 reports the sensitivity of the alternatives.

Range checks identified one additional issue. The sunshine-duration value for 6 April 2024 is recorded as 99. This value is outside the maximum possible daylight duration for Dhaka and appears to be a placeholder. It is retained in the released file in its original form for transparency. It is documented here as a known invalid value and should be treated as missing. The value was excluded from the summary statistics in Table 3.

Quality control was also performed on the individual readings before daily aggregation. The source sheets are maintained manually. They occasionally contain readings that differ from adjacent values. When such an entry was identified, it was repaired by interpolation if the surrounding readings were complete. Otherwise, the daily record was released as missing. The original source sheets are not publicly available. Therefore, users cannot independently verify the individual readings. A small number of daily values may still contain a downward bias from an undetected faulty entry. Isolated single-day anomalies should therefore be checked before analyses that are sensitive to such values.

The four rules differ by up to 740 index units for this date. This shows that the choice of rule affects the estimated value for the missing day. However, its effect on the overall series is negligible. The mean changes by about one index unit, or 0.03%, whether the record is filled using the stated rule or left missing. No statistic, correlation, or comparison reported in this article is materially affected by how this record is treated.

After the data were combined, the weather columns were checked again to make sure they matched the correct demand dates. Range, completeness and consistency checks were also repeated on the final combined data.

The underlying control-room sheets are not publicly available. Therefore, a processing script is provided with the dataset. The script allows the transformation from logged readings to the daily variable to be audited and re-executed. The input table should follow the structure described in Table 7. It should contain one row for each date and one column for each logging time. With this input structure, the script reproduces the released daily series exactly. It also applies the completeness criterion. Any date released as missing is recorded in a log.

#### 4.4.1 Local baseline used for holiday and event deviations

The deviations in Fig. 6 are calculated relative to a local baseline. They are not calculated relative to the annual mean. Using the annual mean could mix event effects with seasonal variation. For example, Victory Day occurs in December. Demand is already lower than the annual average during this period. Therefore, part of the observed deviation could reflect seasonal variation rather than the event itself.

For each date d, the baseline B(d) is calculated using a ± 14-day window around that date. The median daily demand within this window is used as the baseline. Only unaffected days are included in this calculation. These are days with no named event. They are also not marked as public holidays or weekly holidays. The baseline is calculated only when at least five such days are available within the window. Dates with fewer than five days are excluded from Fig. 6.

The deviation for each date is calculated as Δ(d) = (D(d) / B(d) − 1) × 100%, where D(d) is the released daily demand index. For each named event, the plotted value is the mean deviation across all dates associated with that event. The number of contributing dates is shown beside each event label. If a date is associated with more than one event, it is included separately for each event. The baseline is calculated separately for each service area. Each area therefore uses its own demand series. This allows the deviations of Mirpur and Gulshan to be compared directly, even though their overall demand levels are different.

### 4.5 Final dataset assembly and release

All validated variables were combined into the two final daily files. Both files use the same observation period and variable definitions. The same procedures were also used for feature construction, missing-value handling, and quality checks. This consistent processing allows the two area-level series to be compared under the same weather and temporal conditions. The files were then exported as UTF-8 encoded CSV files. They follow the column structure described in Table 2. The files were deposited in the public repository along with the data dictionary.

## Limitations

Several limitations should be considered when reusing these data. First, demand is released only at daily resolution. Hourly values are not covered by the data-sharing permission. Therefore, the data cannot support intraday load-shape analysis or peak-hour forecasting. Second, the released demand variable is an index. It is calculated by summing the load readings recorded for each date. It is expressed in summed megawatts. The source document records the zone load at each logging time. However, it does not state whether each value represents the instantaneous load at that time or the Y maximum load within the interval. The readings also include one supplementary evening-peak value at 19:30. Therefore, the variable is proportional to, but not identical to, daily energy. It should not be interpreted as a metered, billed, or settled energy quantity. Third, the data cover only two distribution service areas in Dhaka. They may not represent electricity consumption in other parts of Bangladesh. Fourth, the residential and commercial labels describe the dominant consumer mix of each service area. They do not represent strictly homogeneous zones. Both areas include a smaller number of other consumer categories. Therefore, comparisons between the two areas represent different area-level consumption contexts rather than pure sectoral demand. Fifth, demand is reported as an area-level aggregate. It is not normalised by the number of consumers, connected load, or floor area. Differences in demand levels may therefore partly reflect differences in service-area size. They should not be interpreted as differences in consumption intensity. Sixth, the meteorological variables were obtained from the Open-Meteo archive. The archive provides gridded reanalysis data rather than in-situ observations from local weather stations. This provides a consistent and gap-free record but may smooth local microclimatic effects. A single reference weather series is also shared by both areas. Therefore, the data cannot capture microclimatic differences between Mirpur and Gulshan. The dataset can be used to study how each area responds to a common weather signal. It cannot be used to study microclimatic differences between the two areas. Seventh, two records are released as missing or invalid rather than observed. These are documented in Section 4.4 and Table 11, Table 12. Finally, the dataset includes demand, weather, and calendar attributes only. Socioeconomic, price, occupancy, and generation-side variables are not included. These factors may also influence electricity consumption. Within these limitations, the dataset provides a consistent and well-documented resource for comparative demand analysis, forecasting, and machine learning.

**Table 12:** Sensitivity of the absent record (16 August 2025) to the choice of replacement rule, and the resulting effect on the series mean. Values are in the units of the daily aggregate demand index.

Replacement rule	Gulshan	Mirpur
Mean of preceding and following day	4628	5142
Mean of the Saturdays within four weeks	4366	5023
Median of the surrounding 29-day window	4825	4997
Janmashtami 2024 deviation applied to the local median	4085	4511
Spread across the four rules	740	631
Series mean if the record is filled (neighbour-mean rule)	3906	4317
Series mean as released, with the record absent	3905	4315

## Credit Author Statement

**MD Sajid Hasan:** Conceptualization, Methodology, Software, Data curation, Formal analysis, Investigation, Visualization, Writing – original draft, Writing – review & editing. **Md. Ashiqur Rahman:** Supervision, Writing – review & editing. **Sanjida Khanom:** Supervision, Formal analysis, Validation, Writing – review & editing. **Mohammod Ashikur Rahman:** Investigation, Validation, Data Curation.

## Ethics Statement

The authors confirm that they have read and followed the ethical requirements for publication in Data in Brief. The work involves no human participants, no animal experiments and no data collected from social media platforms. The electricity demand data were provided by Dhaka Electric Supply Company PLC (DESCO) under written permission dated 22 July 2026, which authorizes academic use, publication and public redistribution of the aggregated daily series described in this article. The meteorological data are obtained from the publicly available Open-Meteo archive and are redistributed in accordance with its terms of use. The dataset contains no personal, confidential or commercially sensitive information: it reports only service-area aggregate daily totals, and no individual consumer, meter, premises or billing information was accessed at any stage. The authors confirm that the data have been prepared and shared in accordance with applicable data-use rules and ethical standards.

## Data Availability

The datasets described in this article are openly available in Mendeley Data at (DOI: 10.17632/3crvdfyvvp.1) https://data.mendeley.com/datasets/3crvdfyvvp/1 under a Creative Commons Attribution 4.0 International (CC BY 4.0) licence. The repository contains both service-area CSV files, a machine-readable data dictionary and a README with the data source, the meteorological retrieval settings, the completeness criterion, the list of records released as missing and the missing-value record. The provenance of the demand variable, its completeness criterion and the aggregation equation are documented in Section 4.1 and Table 7 of this article. The original control-room sheets are not released, because the data-sharing permission covers the aggregated daily series for the two study areas only; they can be made available confidentially to the editorial office on request.

## Declaration of Generative AI and AI-Assisted Technologies in the Manuscript Preparation Process

During the preparation of this work the authors used NotebookLM and ChatGPT.com to improve the language and readability of the manuscript text, at the drafting and revision stages. These tools were not used for data collection, aggregation, quality control, statistical analysis, figure generation or the interpretation of results, all of which were carried out by the authors. After using these tools the authors reviewed and edited the content as needed and take full responsibility for the content of the published article.
